# Δ^24^-Sterol Methyltransferase Plays an Important Role in the Growth and Development of *Sporothrix schenckii* and *Sporothrix brasiliensis*

**DOI:** 10.3389/fmicb.2016.00311

**Published:** 2016-03-11

**Authors:** Luana P. Borba-Santos, Gonzalo Visbal, Thalita Gagini, Anderson M. Rodrigues, Zoilo P. de Camargo, Leila M. Lopes-Bezerra, Kelly Ishida, Wanderley de Souza, Sonia Rozental

**Affiliations:** ^1^Laboratório de Biologia Celular de Fungos, Instituto de Biofísica Carlos Chagas Filho, Universidade Federal do Rio de JaneiroRio de Janeiro, Brazil; ^2^Instituto Nacional de Metrologia, Qualidade e TecnologiaDuque de Caxias, Brazil; ^3^Instituto Venezolano de Investigaciones CientíficasCaracas, Venezuela; ^4^Departamento de Microbiologia, Imunologia e Parasitologia, Universidade Federal de São PauloSão Paulo, Brazil; ^5^Departamento de Biologia Celular, Universidade do Estado do Rio de JaneiroRio de Janeiro, Brazil; ^6^Departamento de Microbiologia, Universidade de São PauloSão Paulo, Brazil; ^7^Laboratório de Ultraestrutura Celular Hertha Meyer, Instituto de Biofísica Carlos Chagas Filho, Universidade Federal do Rio de JaneiroRio de Janeiro, Brazil

**Keywords:** *Sporothrix schenckii*, *Sporothrix brasiliensis*, Δ^24^-sterol methyltransferase, sterol biosynthesis, antifungal activity

## Abstract

Inhibition of Δ^24^-sterol methyltransferase (24-SMT) in *Sporothrix schenckii sensu stricto* and *Sporothrix brasiliensis* was investigated *in vitro*. The effects on fungal growth and sterol composition of the 24-SMT inhibitor 22-hydrazone-imidazolin-2-yl-chol-5-ene-3β-ol (H3) were compared to those of itraconazole. MIC and MFC analysis showed that H3 was more effective than itraconazole against both species in both their filamentous and yeast forms. H3 showed fungistatic activity in a time-kill assay, with inhibitory activity stronger than that of itraconazole. GC analysis of cell sterol composition showed that sterols present in control cells (ergosterol and precursors) were completely replaced by 14α-methylated sterols after H3 exposure. Itraconazole only partially inhibited ergosterol synthesis but completely arrested synthesis of other sterols found in control cells, promoting accumulation of nine 14α-methyl sterols. Based on these results, we propose a schematic model of sterol biosynthesis pathways in *S. schenckii* and *S. brasiliensis*. Effects on cell morphology due to 24-SMT inhibition by H3 as analyzed by SEM and TEM included irregular cell shape, reduced cytoplasmic electron-density, and reduced thickness of the microfibrillar cell wall layer. Moreover, 24-SMT inhibition by H3 promoted mitochondrial disturbance, as demonstrated by alterations in MitoTracker^®^ Red CMXRos fluorescence intensity evaluated by flow cytometry. When used in conjunction with itraconazole, H3 enhanced the effectiveness of itraconazole against all tested strains, reducing at least half (or more) the MIC values of itraconazole. In addition, cytotoxicity assays revealed that H3 was more selective toward these fungi than was itraconazole. Thus, 24-SMT inhibition by H3 was an effective antifungal strategy against *S. schenckii* and *S. brasiliensis*. Inhibition of the methylation reaction catalyzed by 24-SMT has a strong antiproliferative effect via disruption of ergosterol homeostasis, suggesting that this enzyme is a promising target for novel antifungal therapies against sporotrichosis, either as sole treatments or in combination with itraconazole.

## Introduction

In the last decade, the incidence of sporotrichosis, a subcutaneous mycosis with worldwide distribution, has increased in Brazil (Chakrabarti et al., 2015), particularly in the state of Rio de Janeiro (Silva et al., 2012). Moreover, reports of more severe forms of the disease have become increasingly frequent (Almeida-Paes et al., 2014).

Sporotrichosis is caused by dimorphic fungi from the *Sporothrix schenckii* complex (Marimon et al., 2007), with *Sporothrix schenckii sensu stricto* and *Sporothrix brasiliensis* representing the most common clinically isolated forms in Brazil (Rodrigues et al., 2014b) and the most virulent species infecting animal models (Arrigala-Moncrieff et al., 2009; Fernandes et al., 2013). In particular, *S. brasiliensis* is epidemic and responsible for human and feline sporotrichosis cases in the state of Rio de Janeiro, Brazil (Rodrigues et al., 2013).

Itraconazole is the first-choice treatment for cutaneous and lymphocutaneous sporotrichosis (Kauffman et al., 2007). This azole compound inhibits ergosterol biosynthesis at the C14α-demethylation stage (Odds et al., 2003) that is catalyzed by the cytochrome P-450-dependent 14α-demethylase. The resulting ergosterol depletion, with the accumulation of 14α-methylated sterols, interferes with the architecture and fluidity of fungal membranes, which can no longer act as permeability barriers (Odds et al., 2003).

Finding new and more effective agents to treat sporotrichosis is imperative, given the increased incidence and the limitations of the current therapy (itraconazole), which include: (i) the need for long and costly periods of treatment; (ii) the severity of side effects (Kauffman et al., 2007), and (iii) the emergence of isolates with low susceptibility to itraconazole *in vitro* (Rodrigues et al., 2014a; Borba-Santos et al., 2015).

Research efforts have investigated metabolic pathways of pathogenic organisms to find sensitive fungal targets. The search for compounds selective against protozoa and fungi and which spare mammalian cells has led to the development of inhibitors of ergosterol biosynthesis other than at the C14α-demethylase stage. Particular attention has been given to inhibitors of Δ^24^-sterol methyltransferase (24-SMT; an enzyme of ergosterol biosynthesis restricted to plants, protozoa, and fungi) that have shown antimicrobial effects against protozoa and fungi (de Souza and Rodrigues, 2009). However, modulation of 24-SMT activity in *Sporothrix* sp. has not been investigated.

**FIGURE 1:**
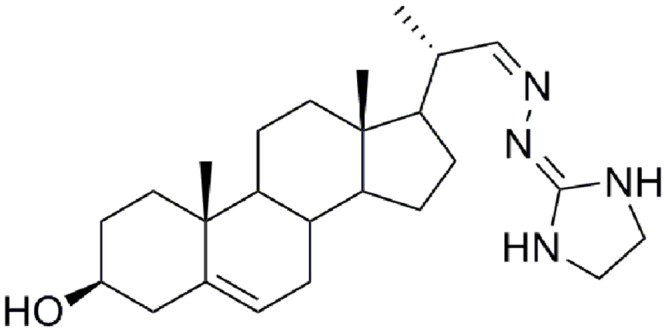
**Molecular structure of the Δ^24^-sterol methyltransferase (24-SMT) inhibitor H3**.

The aim of this study was to determine the effects of 24-SMT inhibition on growth of *S. schenckii sensu stricto* (described as *S. schenckii*) and *S. brasiliensis* and on sterol composition in these fungal species. For this purpose, we used the 24-SMT inhibitor 22-hydrazone-imidazolin-2-yl-chol-5-ene-3β-ol (H3; **Figure 1**). H3 was chosen as it has previously been described as exhibiting anti-proliferative activity against the dimorphic fungus *Paracoccidioides brasiliensis* (Visbal et al., 2011) and the yeasts *Candida albicans* and *Cryptococcus neoformans* (Vivas et al., 2011). Additionally, we compared the effect of H3 to that of itraconazole. We found that inhibition of 24-SMT by H3 completely depleted ergosterol in *S. schenckii* and *S. brasiliensis*, whereas itraconazole only partially blocked its synthesis, pointing to the importance of this enzyme in *Sporothrix* sp. metabolism.

## Materials and Methods

### Fungal Isolates

A total of 32 isolates were used to evaluate the effect of the 24-SMT inhibitor H3: 16 *Sporothrix schenckii sensu stricto* (described here only as *S. schenckii*) isolates (ATCC MYA 4820, ATCC MYA 4821, ATCC 32286, ATCC 16345, Ss-B02, BH1, Ss-B01, Ss 03, Ss 17, Ss 22, Ss 42, Ss 59, Ss 73, Ss116, Ss 119, and Ss 144) and 16 *Sporothrix brasiliensis* isolates (ATCC MYA 4822, ATCC MYA 4823, FMR 8337, Ss 07, Ss 14, Ss 34, Ss 37, Ss 52, Ss 54, Ss 56, Ss 57, Ss 59, Ss 68, Ss 69, Ss 72, and Ss 81), all of which had been previously classified by genotypic identification (Castro et al., 2013; Rodrigues et al., 2013; Borba-Santos et al., 2015). Fungal isolates were stored in potato dextrose agar (PDA; Difco, Detroit, MI, USA) plates at 4°C. For microdilution tests, each strain was grown in the filamentous form in PDA medium at 35°C for 7 days, and the yeast phase was obtained by two successive passages on brain heart infusion broth (Difco, Detroit, MI, USA) supplemented with 2% glucose and incubated at 36°C with orbital agitation (150 rpm) for 7 days.

### Drugs

22-hydrazone-imidazolin-2-yl-chol-5-ene-3β-ol (H3) (**Figure 1**) was synthesized as previously described by Visbal et al. (2011). Itraconazole (Sigma Chemical Co., Saint Louis, MO, USA) was used as a reference antifungal. All drugs were diluted in DMSO to obtains stock solution of 1600 mg/L, and stock solutions were kept at -20°C.

### Antifungal Activity Assays

Microdilution methods based on those found in Clinical and Laboratory Standards Institute (CLSI) documents M27-A3 (yeast form; CLSI, 2008a) and M38-A2 (filamentous form; CLSI, 2008b) and previously described by Borba-Santos et al. (2015) were used to determine the minimum inhibitory concentrations (MIC) of both H3 and itraconazole. The MIC was defined as the lowest concentration of antifungal that inhibits fungal growth relative to untreated controls, as determined by visual inspection in an inverted light microscope, after 5 days of incubation at 35°C in the dark in a humid chamber with 5% CO_2_. Minimum fungicidal concentration (MFC) was determined by plating 10-μL aliquots of fungal samples (from MIC experiments) that had been treated with drug concentrations higher than the MIC onto drug-free PDA plates, which were then incubated at 35°C for 7 days. The MFC was considered the lowest drug concentration that failed to produce fungal growth after 7 days of growth on drug-free PDA.

### Time-Kill Assay and Growth Parameter Determination

*Sporothrix schenckii* strain ATCC MYA 4821 and *S. brasiliensis* strain ATCC MYA 4823, two isolates that had been used in a comparative genomics study (Teixeira et al., 2014) and which will be referred to henceforth as *S. schenckii* 4821 and *S. brasiliensis* 4823, were used as representatives of the two species in time-kill assays as well as in all subsequent experiments. Yeast cells (10^3^ cells/mL) were incubated with 4xMIC or 8xMIC concentrations of itraconazole or H3 in RPMI 1640 medium supplemented with 2% glucose and buffered to pH 7.2 with 0.165 M MOPS for 24, 48, 72, 96, or 168 h at 35°C, after which a 50-μL aliquot of diluted culture was plated on PDA medium and incubated at 35°C for 7 days before colony forming units (CFU) were counted. A reduction in the CFU count of ≥99.9% relative to the starting inoculum was considered fungicidal (Klepser et al., 1998).

The growth kinetics of *S. schenckii* 4821 and *S. brasiliensis* 4823 in RPMI 1640 medium supplemented with 2% glucose and 0.165 M MOPS were determined on untreated controls and used to calculate growth rate (μ) and generation time (g) according to the equations: log_10_N_t_ – log_10_N_0_ = μ(t–t_0_)/2.303 and g = log_e_2/μ, where N_0_ is number of cells at the start of the exponential phase, N_t_ is number of cells at the stationary phase, t_0_ is the starting time and t is the time when the culture reached the stationary phase (Deacon, 2006).

### Extraction and Separation of Neutral Lipids

Total lipids of *S. schenckii* 4821 and *S. brasiliensis* 4823 yeast cells were extracted using the Folch method (Folch et al., 1957). Briefly, 1 mL of extraction solution (chloroform-methanol, 2:1, v/v) for each 1 g of dry extract were added to a glass tube containing yeast untreated or treated for 96 h with sub-inhibitory concentrations (MIC/2) of itraconazole or H3. Samples were lysed mechanically with glass beads (10 cycles of vortex homogenization of 1 min with intervals of 1 min on ice). Suspensions were kept at 4°C for 7 days, and then filtered, concentrated, and suspended with 3 mL of chloroform. Total lipids were applied to a silicic acid column (1.5 cm × 4 cm) and neutral lipids were eluted with four column volumes of chloroform and collected as a single fraction. Solvent was evaporated in a rotary evaporator under vacuum at 50°C, and neutral lipids were suspended and transferred into a 10-mL glass conical centrifuge tube. Samples were dried under nitrogen to be subsequently analyzed by gas chromatography (GC) with mass spectrometry (MS) detection.

### Free Sterol Analyses

For quantitative analysis and structural assignment, neutral lipids were separated in a high-resolution capillary column (Ultra-2, 25 m × 0.20 mm i.d., with 5% phenyl-methylsiloxane and a film thickness of 0.33 μm) in an Agilent Technologies 7890A gas chromatograph equipped with a 5975C inert XL MSD mass selective detector (Agilent Technologies, Inc. USA). Lipids were dissolved in ethyl acetate and injected into the column at an initial temperature of 50°C (1 min), followed by a temperature increase to 270°C at a rate of 20°C/min and a further increase to 290°C at a rate of 1°C/min. The carrier gas (He) flow was kept constant at 1 ml/min. Injector temperature was 250°C and the detector was kept at 280°C. The total run time was 53 min. Mass spectra were obtained by electron ionization (EI) at 70°eV. The assignment of structures was based on relative chromatographic behavior as well as on the characteristic fragmentation patterns observed in MS and by comparison of the mass spectra with those available in the NIST library (http://www.nist.gov/nvl/).

### Transmission Electron Microscopy

*Sporothrix schenckii* 4821 and *S. brasiliensis* 4823 yeast cells were treated for 96 h with sub-inhibitory concentrations (MIC/2) of itraconazole or H3, after which cells were washed in PBS, fixed in 2.5% glutaraldehyde and 4% formaldehyde in 0.1 M cacodylate buffer (pH 7.2) for 24 h at 4°C, and then post-fixed in 1% osmium tetroxide in 0.1 M cacodylate buffer containing 1.25% potassium ferrocyanide and 5 mM CaCl_2_ for 2 h at 4°C. Cells were washed, dehydrated in a series of ethanol solutions of increasing concentration (30, 50, 70, 90, 100%, and ultra-dry ethanol) for 30 min at each step, and then embedded in Spurr resin. Ultrathin sections were stained in uranyl acetate and lead citrate and observed in a JEOL 1200 EX electron microscope (JEOL Ltd., Japan) equipped with a CCD camera Megaview III (Soft Image System, Germany). Images were acquired using iTEM software (Soft Image System, Germany). ImageJ software (NIH, USA) was used to determine the thickness of the inner cell wall (ICW) and of the outer microfibrillar layer (ML) of the cell wall in 20 cells per sample.

### Scanning Electron Microscopy

*Sporothrix schenckii* 4821 and *S. brasiliensis* 4823 yeast cells treated for 96 h with sub-inhibitory concentrations (MIC/2) of itraconazole or H3 were fixed as described above (see “Transmission Electron Microscopy”), after which 100-μL aliquots of each sample were adhered to poly-L-lysine-coated glass coverslips, post-fixed with 1% osmium tetroxide in 0.1 M cacodylate buffer containing 1.25% potassium ferrocyanide, dehydrated in a graded ethanol series, critical-point-dried in CO_2_, and coated with gold. Images were obtained in a FEI Quanta 250 scanning electron microscope (FEI Company, USA). In order to determine cell size, mean Feret diameter (distance between two parallel lines tangent to the 2D projection of a 3D object) was calculated for 50 yeast-like cells using Image J software (NHI, USA). Shapes of yeast-like cells were determined according aspect ratio (ratio between maximum and minimum Feret diameters), wherein values closer to 1 indicate a globose/oval morphology and values much higher or lower than 1 indicate an elongated morphology.

### Flow Cytometry Analysis

*Sporothrix schenckii* 4821 and *S. brasiliensis* 4823 yeast cells treated for 96 h with sub-inhibitory concentrations (MIC/2) of itraconazole or H3 (10^6^ cells/mL) were washed in PBS and incubated with 10 μM of MitoTracker^®^ Red CMXRos (Molecular Probes^TM^, USA) for 30 min at room temperature in the dark, after which the cells were washed in PBS, fixed in 1% formaldehyde in PBS and washed again. Samples were analyzed in a BD Accuri^TM^ C6 flow cytometer (BD Biosciences, USA) that counted 2000 events per sample, and data were analyzed using BD Accuri C6 software. Results are representative of three independent experiments. MitoTracker^®^ Red CMXRos stains mitochondria in live cells, and its accumulation is dependent upon membrane potential.

### Drug Interaction Assay

A checkerboard microdilution method (Pillai et al., 2005) was used to examine interactions between itraconazole and H3, using four isolates from each species in the yeast form. Itraconazole and H3 were tested at concentrations of 0.06–4 mg/L and 0.03–0.5 mg/L (in serial 1:2 dilutions), respectively, against final yeast concentrations of 0.5–2.5 × 10^3^CFU/mL at 35°C for 5 days. Antifungal combinations were classified according the fractional inhibitory concentration index (FICI), defined by the equation: FICI = (MICa in combination/MICa tested alone) + (MICb in combination/MICb tested alone), where “a” was itraconazole and “b” was H3 (Pillai et al., 2005). Interactions were considered synergistic if FICI ≤ 0.5, absent if FICI >0.5 and ≤ 4, and antagonistic if FICI >4 (Odds, 2003).

### Drug Selectivity Toward Fungal Cells

To evaluate the selectivity of H3 toward *S. schenckii* and *S. brasiliensis*, the concentration of this compound that elicited 50% cytotoxicity (CC_50_) toward monkey cell line LLC-MK2 cells or 50% hemolysis (HA_50_) of human red blood cells was estimated as described previously (Ishida et al., 2006). Selectivity indexes were calculated according to the formula: SI = CC_50_ or HA_50_/median MIC. For comparison with H3, we also determined CC_50_ and HA_50_ values for itraconazole in parallel.

### Statistical Analysis

Statistical analysis was performed using Prism 5.0 (GraphPad Software, Inc., La Jolla, CA, USA), and statistical significance was accepted when *p* < 0.05. The Mann–Whitney test was used to analyze differences between antifungal treatments. Differences in cell size and in the thickness of cell wall components between untreated and treated yeast cells were analyzed by one-way ANOVA (Dunnett’s test).

## Results

### The 24-SMT Inhibitor H3 is more Effective than is Itraconazole against *S. schenckii* and *S. brasiliensis*

To evaluate the sensitivity of *S. schenckii* and *S. brasiliensis* isolates to the 24-SMT inhibitor H3 (a sterol hydrazone analog), the MIC and MFC of H3 and of itraconazole were determined *in vitro* for filamentous and yeast forms. The observed MICs of H3 were lower than those of itraconazole (*p* < 0.0001), as were its MFC mode values, indicating that H3 was the more effective compound against both species (**Table 1**). Itraconazole was less effective against *S. brasiliensis* than it was against *S. schenckii* (*p* = 0.0015 and 0.008 for the filamentous and yeast forms, respectively).

**Table 1:** Antifungal activity of 24-SMT inhibitor H3 and itraconazole against *Sporothrix schenckii* and *Sporothrix brasiliensis.*

		Filamentous form				Yeast form			
Species	Compound	MIC range^a^	MIC mode^b^	MFC range^c^	MFC mode^d^	MIC range^a^	MIC mode^b^	MFC range^c^	MFC mode^d^
*S. schenckii*	H3	0.01–0.5	0.25	0.03–8	1	0.03–0.25	0.125	0.06–8	0.25
(*n* = 16)	Itraconazole	0.06–1	0.5	0.06 – >16	>16	0.06–1	0.5	0.25 – >16	0.5
*S. brasiliensis*	H3	0.03–0.5	0.25	0.25 – >16	8	0.01–0.25	0.125	0.03–16	0.125
(n = 16)	Itraconazole	0.25 – > 16	4	0.25 – >16	>16	0.25–4	0.5	0.25– >16	0.5

*All values are expressed in mg/L. ^a^Range of MIC values (range of lowest drug concentrations that prevents fungal growth relative to untreated controls) for the different fungal strains tested. ^b^Mode of MIC values (MIC values that occur most frequently). ^c^Range of MFC values (range of lowest drug concentrations that produced no fungal growth) for the fungal isolates tested. ^d^Mode of MFC values (MFC values that occur most frequently).*

Data are displayed as mean and standard error of the mean and are representative of two independent experiments made in duplicate.

### H3 has Fungistatic Activity against *S. schenckii* and *S. brasiliensis*

**FIGURE 2:**
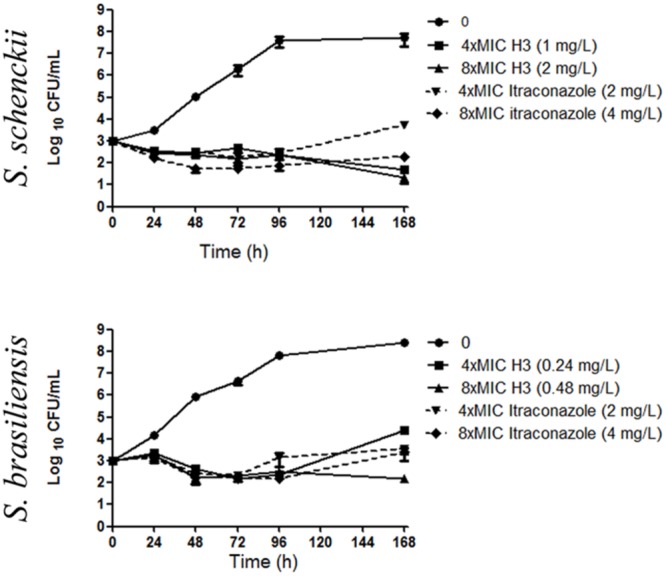
**Time-kill plots showing activity of the 24-SMT inhibitor H3 in comparison with that of itraconazole against the yeast forms of *Sporothrix schenckii* ATCC MYA 4821 and *S. brasiliensis* ATCC MYA 4823**.

To determine whether the activity of H3 on *S. schenckii* and *S. brasiliensis* was fungicidal or fungistatic, we performed time-kill assays using *S. schenckii* 4821 and *S. brasiliensis* 4823 isolates (**Figure 2**). Time-kill assays for itraconazole were performed in parallel (**Figure 2**). H3 had fungistatic activity against both isolates. For *S. schenckii* 4821, the inhibitory activity of H3 was stronger than that of itraconazole: after 168 h, there was a 2 Log_10_ reduction in yeast viability for H3 at 4xMIC, compared with a reduction of 1 Log_10_ for itraconazole at 8xMIC. For *S. brasiliensis* 4823, the inhibitory activity of H3 was slightly stronger than that of itraconazole, with a reduction in yeast viability of 1 Log_10_ for H3 at 8xMIC.

Growth curve data (**Figure 2**, untreated samples) indicate that both species reached the stationary phase after 96 h of growth at 35°C, with a generation time (g) of 7 h and 27 min and a growth rate (μ) of 0.093/h. Therefore, this time-point was chosen for treatment with H3 or itraconazole in the subsequent experiments.

### Sterols Found in *S. schenckii* and *S. brasiliensis*

The free sterol composition of *S. schenckii* and *S. brasiliensis* as measured by high-resolution capillary gas chromatography coupled to mass spectrometry (GC-MS) is presented here in detail for the first time in the literature (**Table 2**). Although ergosterol was the major sterol, representing over 83% of the total amount of sterols, some more unusual sterols were also detected as minor sterol components in *S. schenckii* 4821 and *S. brasiliensis* 4823, including: (i) ergosta-5,7,22,24(24′)-tetraen-3β-ol; (ii) ergosta-5,7-dien-3β-ol/5-dehydro-episterol, which co-eluted at 26.6 min; and (iii) stigmasterol (**Table 2**).

**Table 2:** Free sterols present in *Sporothrix schenckii* ATCC MYA 4821 and *Sporothrix brasilensis* ATCC MYA 4823 growth in untreated (control) and treated with H3 o itraconazole.

		*Sporothrix schenckii*			*Sporothrix brasiliensis*		
Sterol	Retention Time in (min)	Control	H3	Itraconazol	Control	H3	Itraconazol
14α-Methyl-5α-cholesta-8-en-3-one (**1**)^∗^	24.4	–	–	–	–	7.3	–
Ergosterol (**2**)^∗^	25.5	83.1	–	31.2	92.1	–	33.7
14α-Methyl-5α-ergosta-8,24(24′)-dien-3β-ol (**3**)^∗^	25.8	–	–	3.1	–	24.1	4.8
Ergosta-5,7,22,24(24′)-tetraen-3β-ol (**4**)^∗^	25.9	3.9	–	–	–	–	–
Ergosta-5,7,dien-3β-ol (**5**)/5-Dehydro-episterol (**6**)^∗^	26.6	7.7	–	–	4.7	–	–
4,14-Dimethyl-ergosta-5,7-24(24′)-trien-3β-ol (**7**)^∗^	26.7	–	–	4.5	–	–	3.3
Stigmasterol (**8**)^∗^	27.2	5.2	–	–	3.2	–	–
Obtusifoliol (**9**)^∗^	27.3	–	–	15.6	–	–	21.5
Lanosta-8,24-dien-3-one (**10**)^∗^	27.6	–	30.1	10.0	–	4.5	6.0
Lanosterol (**11**)^∗^	28.4	–	69.8	–	–	64.1	–
24-Methylene-lanosta-8-en-3-one (**12**)^∗^	29.2	–	–	4.4	–	–	–
Eburicol (**13**)^∗^	30.0	–	–	17.4	–	–	20.1
24-Ethyl-lanosta-8,22-dien-3β-ol (**14**)^∗^	32.3	–	–	2.1	–	–	–
(*E*)24-ethylidenelanost-8-en-3β-ol (**15**)^∗^	32.8	–	–	4.1	–	–	3.8
(*Z*)24-ethylidenelanost-8-en-3β-ol (**16**)^∗^	33.3	–	–	7.4	–	–	6.7

*^∗^Numbers in parenthesis correspond to the numbers used in the schematic model **Figure 6**.*

### Inhibition of 24-SMT by H3 Leads to Ergosterol Depletion in *S. schenckii* and *S. brasiliensis*

Analyses of the free sterol composition of cells after treatment with sub-inhibitory concentrations (MIC/2) of the 24-SMT inhibitor H3 revealed that the sterols present in control cells (ergosterol and precursors) were completely replaced by 14α-methylated sterols. Both *S. brasiliensis* 4823 and *S. schenckii* 4821 accumulated mainly lanosterol (between 64.1 and 69.8%) (**Table 2**), pointing to a significant perturbation of the C-24 alkylation reaction corresponding to 24-SMT inhibition. However, there were differences between the two species in the extent to which other intermediates were accumulated. The rate of accumulation of lanosta-8,24-dien-3-one was 30.2% in *S. schenckii* 4821 and 4.5% in *S. brasiliensis* 4823, which also accumulated two other intermediates not found in *S. schenckii* 4821: 14α-methyl-ergosta-8,24(24′)-dien-3β-ol (24.1%) and 14α-methyl-cholesta-8-en-3-one (7.3%) (**Table 2**). The accumulation of 3-ketosteroids (14α-Methyl-5α-cholesta-8-en-3-one and lanosta-8,24-dien-3-one) indicates that H3 also interferes directly or indirectly with the NADPH-dependent 3-ketosteroid reductase.

### Itraconazole does not Completely Block Ergosterol Synthesis in *S. schenckii* and *S. brasiliensis*

Itraconazole only partially inhibited ergosterol synthesis (∼63%) but completely arrested synthesis of other sterols (ergosta-5,7,22,24(24′)-tetraen-3β-ol, ergosta-5,7,dien-3β-ol/5-dehydro-episterol, and stigmasterol) which were found in control cells (**Table 2**). Likewise, sterol analysis revealed that both itraconazole treated isolates accumulated nine 14α-methyl sterols: (i) obtusifoliol, (ii) lanosta-8,24-dien-3-one, (iii) eburicol, (iv) 14α-methyl-ergosta-8,24(28)-dien-3β-o, (v) 4,14-dimethyl-ergosta-5,7-24(28)-trien-3β-ol, (vi) 24-methylene-lanosta-8-en-3-one, (vii) 24-ethyl-lanosta-8,22-dien-3β-ol, (viii) 24(*E*)-ethylidenelanosta-8-en-3β-ol, and (ix) 24(*Z*)-ethylidenela nosta-8-en-3β-ol (**Table 2**).

### Inhibition of 24-SMT Changes the Morphology of *S. schenckii* and *S. brasiliensis*

**FIGURE 3:**
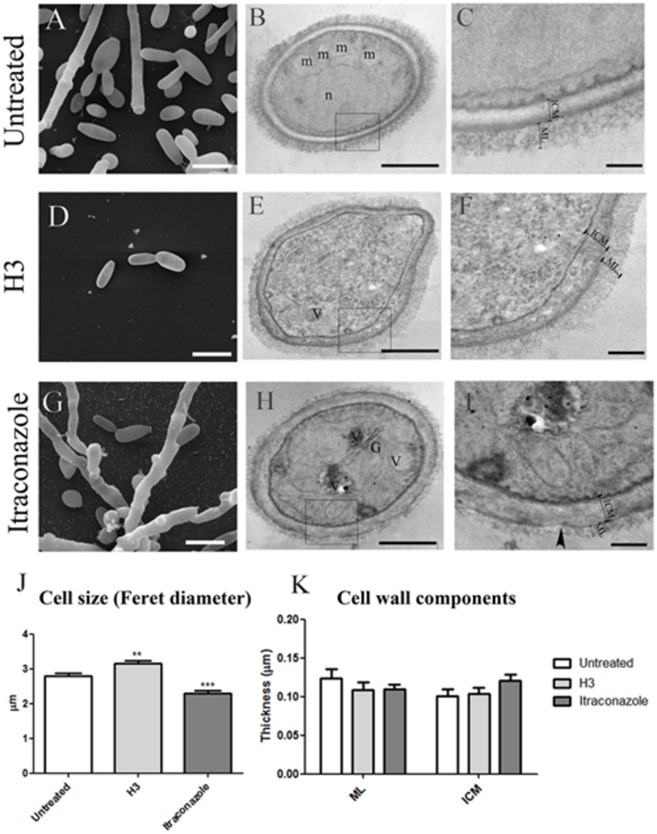
**Ultrastructural analysis of *Sporothrix schenckii* ATCC MYA 4821 yeast cells treated with 0.125 mg/L H3 (D–F) or 0.25 mg/L itraconazole (G–I) (MIC/2) compared to untreated cells **(A–C)**.** Scanning electron microscopy **(A,D,G)** and transmission electron microscopy **(B,C,E,F,H,I)** images show the presence of: m, mitochondria **(B)**; n, nucleus **(B)**; G, Golgi complex **(H)**; inner cell wall (ICW); microfibrillar cell wall layer (ML). The arrowhead in **(I)** indicates a groove in the cell wall structure. **(J)** Cell diameter analysis by calculation of Feret diameters. **(K)** Analysis of ICW and ML thickness as measured in TEM images. ^∗∗^*p* < 0.001; ^∗∗∗^*p* < 0.0001. Scale bars: 5 μm **(A,D,G)**; 0.5 μm **(B,E,H)**; 0.2 μm **(C,F,I)**.

**FIGURE 4:**
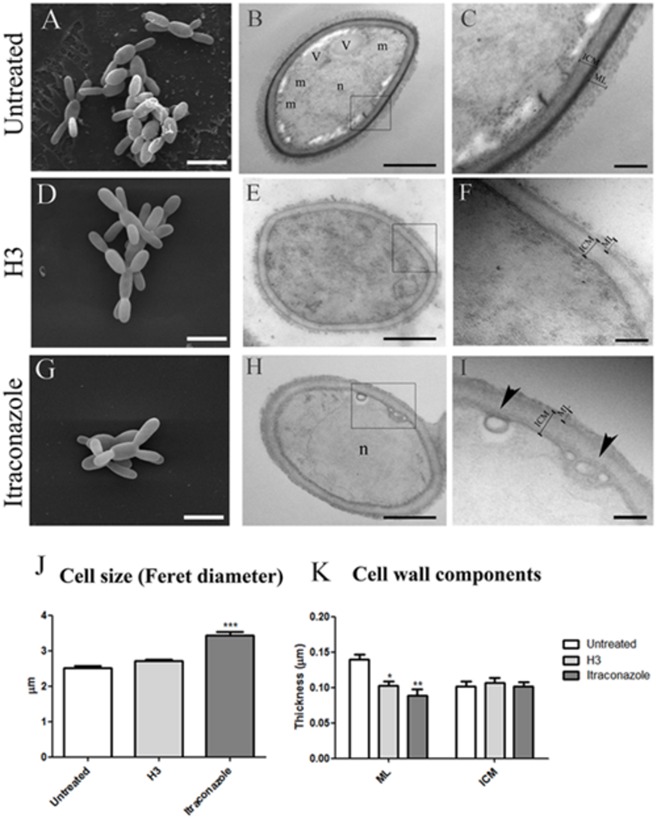
**Ultrastructural analysis of *Sporothrix brasiliensis* ATCC MYA 4823 cells after exposure to 0.03 mg/L H3 **(D–F)** or 0.25 mg/L itraconazole **(G–I)** compared to untreated cells **(A–C)**.** Scanning electron microscopy **(A,D,G)** and transmission electron microscopy **(B,C,E,F,H,I)** images show the presence of: m, mitochondria **(B)**; n, nucleus **(B,H)**; v, vacuole **(B)**; inner cell wall (ICW); microfibrillar cell wall layer (ML). Arrowheads indicate small vesicles associated with the plasma membrane **(I)**. **(J)** Cell diameter analysis by calculation of Feret diameters. **(K)** Analysis of ICW and ML thickness as measured in TEM images. ^∗^*p* < 0.05, ^∗∗^*p* < 0.001, ^∗∗∗^*p* < 0.0001. Scale bars: 5 μm **(A,D,G)**; 0.5 μm **(B,E,H)**; 0.2 μm **(C,F,I)**.

To determine the effect of 24-SMT inhibition on cell morphology, *S. schenckii* 4821 and *S. brasiliensis* 4823 yeast cells were analyzed by scanning and transmission electron microscopy (SEM and TEM, respectively) after treatment with sub-inhibitory concentrations (MIC/2) of H3 for 96 h (**Figures 3** and **4**). The same cells treated with itraconazole were similarly analyzed.

SEM analysis revealed that untreated yeast cells of both isolates had an elongated shape (aspect ratio medians were 1.83 and 1.70 for *S. schenckii* 4821 and *S. brasiliensis* 4823 cells, respectively) (**Figures 3A** and **4A**). Measurement of Feret diameters revealed that *S. schenckii* 4821 yeast cells were larger than those from *S. brasiliensis* 4823 (*p* = 0.048). Yeast-hyphae conversion was observed in *S. schenckii* 4821 control and itraconazole treated cultures but not in H3 treated cultures (**Figures 3A,D,G**). In addition, we observed an increase in cell size (*p* < 0.001, **Figure 4J**) after treatment of *S. schenckii* with H3 and a decrease in cell size after itraconazole treatment (*p* < 0.001, **Figure 4J**). In contrast, treatment of *S. brasiliensis* 4823 with itraconazole led to an increase in cell size (*p* < 0.0001, **Figure 4J**).

TEM images of untreated *S. schenckii* 4821 (**Figures 3B** and **4C**) and *S. brasiliensis* 4823 (**Figures 4B,C**) cells revealed an electron-dense cytoplasm containing mitochondria and a nucleus surrounded by a cell membrane and a cell wall with a clear inner layer (ICW) and a compact microfibrillar outer layer (ML). The outer layer was thicker in *S. brasiliensis* 4823 than in *S. schenckii* 4821 (*p* = 0.017).

*Sporothrix schenckii* 4821 cells treated with H3 showed reduced cytoplasmic electron-density (**Figure 3E**), while itraconazole treatment promoted accumulation of electron-dense vacuoles next to the Golgi complex and electron-lucent vacuoles in the cytoplasm (**Figure 3H**) while also inducing grooves in the cell wall (arrow in **Figure 3I**). No statistically significant differences in the thickness of cell wall components were detected between control and treated cells (**Figure 3K**).

Treatment of *S. brasiliensis* 4823 cells with H3 decreased ML thickness (*p* < 0.001) (**Figure 4K**) but did not result in clear changes in cytoplasmic ultrastructure (**Figures 4E,F**). However, treatment with itraconazole reduced cytoplasmic electron-density in *S. brasiliensis* 4823 and was associated with the presence of small vesicles next to the plasma membrane (**Figures 4H,I**, arrows) and with decreased ML thickness (*p* < 0.001) (**Figure 4K**).

### Inhibition of 24-SMT Induces Mitochondrial Disturbances in *S. schenckii* and *S. brasiliensis*

**FIGURE 5:**
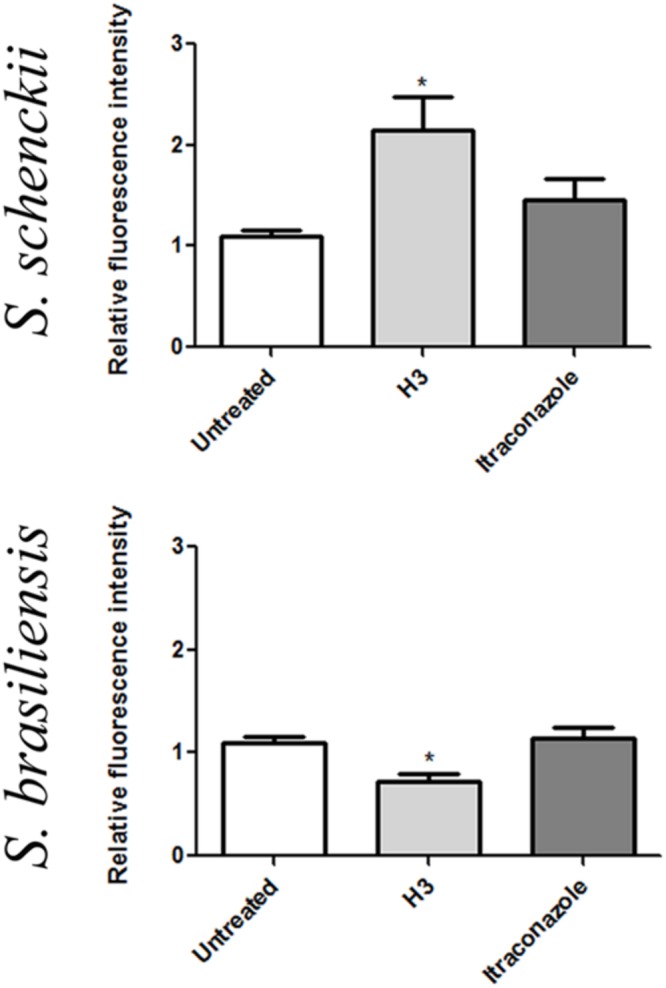
**Mitochondrial activity after treatment of *Sporothrix schenckii* ATCC MYA 4821 and *S. brasiliensis* ATCC MYA 4823 yeast cells with H3 or itraconazole.** Cells untreated or treated for 96 h with sub-inhibitory concentrations of H3 or itraconazole were stained with MitoTracker Red CMXRos, after which fluorescence intensity was analyzed by flow cytometry. H3 exposure induced a statistically significant fluorescence intensity increase (*S. schenckii* 4821) or decrease (*S. brasiliensis* 4823), reflecting mitochondrial disturbance (^∗^*p* < 0.05).

Mitochondria play a central role in maintaining homeostasis of the cell. Mitochondrial activity was evaluated after exposure to H3 or to itraconazole by MitoTracker Red^®^ CMXRos stain and flow cytometry (**Figure 5**). H3 exposure promoted mitochondrial disturbances in both isolates, as demonstrated by the increase (*S. schenckii* 4821) and decrease (*S. brasiliensis* 4823) in MitoTracker Red CMXRos fluorescence intensity of stained cell populations (*p* < 0.05) (**Figure 5**). In contrast, no statistically significant change in mitochondrial activity was observed after itraconazole exposure (**Figure 5**).

### H3 Increases the Effectiveness of Itraconazole against *S. schenckii* and *S. brasiliensis*

In view of the high effectiveness of H3, we decided to test whether H3 could improve itraconazole activity. Thus, checkerboard microdilution assays were performed using the yeast forms of four isolates from each species in order to determine whether itraconazole and H3 act synergistically. Co-incubation led to a reduction in the itraconazole MIC for all tested strains (**Table 3**). However, the interaction between H3 and itraconazole was considered synergistic only for the *S. brasiliensis* strains 4823 and Ss 37 (FICI = 0.5 and 0.27, respectively).

**Table 3:** Associations of itraconazole and H3 against *Sporothrix schenckii* and *Sporothrix brasiliensis* yeast cells. Minimum inhibitory concentration (MIC) values are in mg/L.

Species	Fungal isolate	MIC drugs tested alone		MIC drugs in combination		
		H3	Itraconazole	H3	Itraconazole	FICI^a^
*S. schenckii*	4821	0.06	0.5	0.06	0.25	1.50
	4820	0.25	0.5	0.06	0.25	0.74
	Ss 03	0.25	0.25	0.125	0.125	1.00
	Ss 42	0.125	0.25	0.125	0.03	0.74
*S. brasiliensis*	4823	0.06	0.5	0.03	0.03	0.50^b^
	Ss 14	0.03	2.0	0.03	0.06	1.00
	Ss 37	0.125	2.0	0.03	0.06	0.27^b^
	Ss 69	0.06	0.5	0.06	0.25	1.50

*^a^Fractional inhibitory concentration index, FICI = (MICa in combination/MICa tested alone) + (MICb in combination/MICb tested alone), where a and b are itraconazole and H3, respectively.*

*^b^Synergistic interactions (FICI ≤ 0.5).*

### H3 Exhibits Great Selectivity toward *S. schenckii* and *S. brasiliensis*

To estimate the selectivity of H3 toward *S. schenckii* and *S. brasiliensis*, we determined the cytotoxic effect of this compound (and also of itraconazole) in LLC-MK2 cells (CC_50_) as well as their hemolytic activity (HA_50_) (**Table 4**). The CC_50_ of H3 was similar to that of itraconazole. No hemolytic effect toward red blood cells was observed for either of the tested drugs at concentrations up to 100 mg/L. Analysis of the selectivity index (CC_50_ or HA_50_/median MIC) revealed that H3 was at least 666 times more selective toward both yeast and filamentous forms of *S. schenckii* and *S. brasiliensis* than it was toward LLC-MK2 and red blood cells (**Table 4**).

**Table 4:** Selectivity of 24-SMT inhibitor H3, compared to itraconazole, toward *Sporothrix schenckii* and *Sporothrix brasiliensis* yeast cells.

Compound	Yeast MIC^a^ medians (mg/L)		Cytotoxicity against LLC-MK2 cells			Hemolytic activity against red blood cells		
	*S. schenckii*	*S. brasiliensis*	CC_50_^b^ (mg/L)	SI^c^ *S. schenckii*	SI^c^ *S. brasiliensis*	HA_50_^d^ (mg/L)	SI^c^ *S. schenckii*	SI^c^ *S. brasiliensis*
H3	0.15	0.1	100	666.7	1000	>100	>666.7	>1000
Itraconazole	0.39	1.17	>100	>256.4	>85.5	>100	>256.4	>85.5

*^a^Minimum inhibitory concentration, ^b^50% cytotoxic concentration, ^c^Selective index: SI = CC_50_ or HA_50_/MIC medians, ^d^50% hemolytic concentration.*

## Discussion

We investigated the effect of the 24-SMT inhibitor H3 (a sterol hydrazone analog) on the sterol biosynthesis pathway in *S. schenckii* and *S. brasiliensis.* We found that H3 was more effective than was itraconazole (currently the first choice for treatment of sporotrichosis) in arresting the growth of *Sporothrix* sp. The H3 MICs obtained for *S. schenckii* and *S. brasiliensis* (MIC mode ≤ 0.25 mg/L; **Table 1**) were similar to those previously reported for *P. brasiliensis* (1 μM, corresponding to 0.4 mg/L) (Visbal et al., 2011). In contrast, H3 has been reported to be less active against some pathogenic yeasts, including *Candida albicans, Candida parapsilosis, Cryptococcus neoformans* and *Cryptococcus gattii* (MIC values around 4 mg/L) (Vivas et al., 2011).

In addition, the MIC and time-kill results showed that itraconazole was less active *in vitro* against *S. brasiliensis* than it was against *S. schenckii*, a surprising finding. This difference in activity between species was not observed for H3 (**Table 1**; **Figure 2**).

**FIGURE 6:**
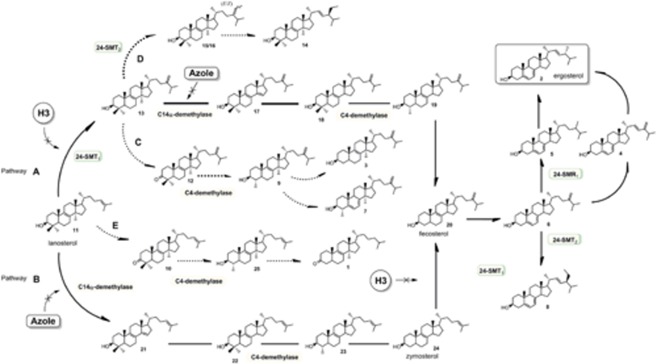
**Representative pathway of sterol biosynthesis in *Sporothrix schenckii* and *Sporothrix brasiliensis*, and sites of action of the studied inhibitors.** Sterols identified on **Table 2** are shown in this figure: **(1)** 14α-methyl-cholesta-8-en-3-one, **(2)** ergosterol, **(3)** 14α-methyl-ergosta-8,24(24’)-dien-3β-ol, **(4)** ergosta-5,7,22,24-tetra-en-3β-ol, **(5)** ergosta-5,7,24(24′)-trien-3β-ol, **(6)** 5-dehydro-episterol, **(7)** 4,14-dimethyl-ergosta-5,7,24(24’)-trien-3β-ol, **(8)** stigmasterol, **(9)** obtusifoliol, **(10)** lanosta-8,24-dien-3-one, **(11)** lanosterol, **(12)** 24-methylene-lanosta-8-en-3-one, **(13)** eburicol, **(14)** 24-ethyl-lanosta-8,22-dien-3β-ol, **(15)** 24(*E*)-ethylidenelanost-8-en-3β -ol, **(16)** 24(*Z*)-ethylidenelanost-8-en-3β-ol, **(17)** 4,4-dimethyl-ergosta-8,14,24(24′)-trien-3β-ol, **(18)** 4,4′-dimethyl-ergosta-8,24(24′)-dien-3β-ol, **(19)** 4-methyl-ergosta-8,24(24′)-dien-3β-ol, **(20)** fecosterol, **(21)** 4,4-dimethyl-ergosta-8,14,24-trien-3β-ol, **(22)** 4,4-dimethyl-ergosta-8,24-dien-3β-ol, **(23)** 4-methyl-ergosta-8,24-dien-3β-ol, and **(24)** zymosterol. Thick lines indicate the main pathways (pathway A or B) from lanosterol to ergosterol. Dashed arrows show accumulation of intermediate sterols by action of the sterol biosynthesis inhibitors itraconazole and H3 (pathways C, D, E). Arrows marked with an ‘X’ indicate inhibitory interactions of the sterol hydrazone H3 and itraconazole with Δ^24^-sterol methyl transferase (24-SMT_1_) and C14α-demethylase, respectively. 24-SMR, Δ^24^-sterol methyl reductase.

The sterol biosynthesis pathway in some fungi is well established, and two main ergosterol biosynthesis pathways are known (pathways A and B, **Figure 6**), but little is known about the pathway of ergosterol biosynthesis in *Sporothrix* sp. Therefore, the use of sterol biosynthesis inhibitors (such as those used here) that lead to accumulation of sterol intermediates may reveal novel metabolic pathways.

In its early stages, fungal ergosterol synthesis uses an isoprenoid pathway to squalene oxide similar to the one involved in cholesterol formation (Mercer, 1993; Nes, 2011). In most fungi, the key transformations from lanosterol to ergosterol take place in the following order: (i) introduction of a methyl group into lanosterol at C-24, accompanied by a double-bond shift from Δ^24(25)^ to Δ^24(24′)^ to yield eburicol; (ii) demethylation at C-14 to yield a 4,4-dimethyl-Δ^8,14^-sterol; (iii) saturation of the -Δ^14^ double bond introduced in the previous step to yield 4,4-dimethyl-Δ^8^-sterol; (iv) successive removal of the two methyl groups at C-4 to yield fecosterol; (v) a double bond shift from Δ^8^ to Δ^7^ and introduction of a double bond at Δ^5^ to yield ergosta-5,7,24(24′)-trien-3β-ol; (vi) introduction of a double bond at Δ^22^ to yield ergosta-5,7,22,24(24′)-tetraen-3β-ol; and reduction of the Δ^24(24′)^ double bond to yield ergosterol (pathway A, **Figure 6**). Other fungi such as *Saccharomyces cerevisiae* and *C. albicans* constitute important exceptions, in which lanosterol is demethylated in the nucleus at C-4 and C-14 before C-24 alkylation of zymosterol (pathway B, **Figure 6**) (Song and Nes, 2007).

As is true for most pathogenic fungi, the major sterol detected in *S. schenckii* and *S. brasiliensis* was ergosterol (**Table 2**). Nevertheless, less usual sterols were also observed to a lesser degree, including ergosta-5,7,22,24(24′)-tetraen-3β-ol, ergosta-5,7-dien-3β-ol, 5-dehydro-episterol, and stigmasterol (**Table 2**). An interesting finding was the identification of C-24 ethyl sterols in *S. brasiliensis* that are more usual in plant cells, where they have multiple roles related to plant cell growth and development (Nes et al., 2006). However, few reports exist describing the detection in fungi of 24-ethyl sterols, for which no role has been assigned yet. Based on the results of the present study, we propose a schematic model of sterol biosynthesis pathways in *S. schenckii* and *S. brasiliensis* (**Figure 6**), showing the sites of action of the studied inhibitors.

*Sporothrix schenckii* 4821 and *S. brasiliensis* 4823 grown in the presence of itraconazole accumulated 14α-methylated sterols (**Table 2**, see number 3, 7, 9, 10, 12, to 16), which account for more than 60% of the total sterols. Ergosterol is still detected and corresponds to about 30% of total sterols. These results indicate that, at sub-inhibitory concentrations, itraconazole only partially blocks ergosterol synthesis. Accumulation of eburicol, obtusifoliol, and lanosta-8,24-dien-3-one indicates that C14α-demethylase was inhibited. The introduction of a methyl group into lanosterol at C-24, accompanied by a double-bond shift from Δ^24(25)^ to Δ^24(24′)^ to yield eburicol and obtusifoliol, strongly suggests that C-24 alkylation occurs as the first step in the conversion of lanosterol to ergosterol in these fungi (pathway A, **Figure 6**). This result is consistent with a previous study on *P. brasiliensis* (Visbal et al., 2011). Accumulation of lanosterol-8,24-dien-3-one, 24-methylene-lanosta-8-en-3-one, and eburicol may indicate that the presence of a 14α-methyl group blocks the first C4α-demethylation process. Although this blockage would not be complete as shown by the biosynthesis of the sterols 4,14-dimethyl-ergosta-5,7-24(24′)-trien-3β-ol and obtusifoliol (pathway C, **Figure 6**). The second C4α-demethylation process seems to be more inhibited, based on the presence of small amounts of 14α-methyl-5α-ergosta-8,24(24′)-dien-3β-ol (pathway C, **Figure 6**). Another possibility is that itraconazole inhibits not only C14α-demethylase but also NADPH-dependent 3-ketosteroid reductase, i.e., the enzyme catalyzing the last step in the demethylation at C-4 (Vanden Bossche et al., 1993; Marichal et al., 1999). This inhibition may occur directly or indirectly.

Three other uncommon lanostane-type triterpenoids were identified in fungi grown in the presence of itraconazole: 24(*E*)-ethylidenelanosta-8-en-3β-ol, 24(*Z*)-ethylidenelanosta-8-en-3β-ol, and 24-ethyl-lanosta-8,22-dien-3β-ol (**Table 2**). This data indicates that lanosterol is not the only substrate of 24-SMT (Kaneshiro et al., 1999b), as eburicol, which can accept a second methyl group on the Δ^24(24′)^-bond (pathway D, **Figure 6**), is also a substrate, albeit a poorer one. Previous reports of these sterols from human-derived *P. carinii* (Kaneshiro et al., 1999a,b; Amit and Kaneshiro, 2001) helped to assign the correct configuration of the isomers Z and E on the side chain of 24(*E*)-ethylidenelanost-8-en-3β-ol and 24(*Z*)-ethylidenelanost-8-en-3β-ol. The accumulation of 24-ethylsterols in *Sporothrix* sp. exposed to itraconazole suggests that 24-SMT activities are very high in these organisms.

In contrast, ergosterol synthesis was completely blocked in *S. schenckii* and *S. brasiliensis* grown in the presence of the 24-SMT inhibitor H3, resulting in a strong increase in the levels of lanosterol (64–70%, **Table 2**). This result confirms that lanosterol is first converted into eburicol by 24-SMT and then undergoes C14α-demethylation (pathway A, **Figure 6**). Hence, we can conclude that H3 inhibits fungal growth by interfering with 24-SMT, as has been previously reported in *P. brasiliensis* (Visbal et al., 2011).

*Sporothrix brasiliensis* treated with H3 also accumulated two 3-ketosteroids (14α-methyl-5α-cholesta-8-en-3-one and lanosta-8,24-dien-3-one), suggesting that H3 allows the eburicol biosynthesis followed by demethylation at C-4, with little interference in the activity of NADPH-dependent 3-ketosteroid reductase (pathway C, **Figure 6**). On the other hand, *S. schenckii* accumulated a higher proportion of lanosta-8,24-dien-3-one (pathway E, **Figure 6**), which indicates that the NADPH-dependent 3-ketosteroid reductase of *S. schenckii* was more susceptible to the action of H3. Accumulation of 3-ketosteroids can contribute positively to the antifungal activity of H3 in *Sporothrix* sp. because these sterols have been shown to destabilize the lipid bilayer and to inhibit the growth of sterol-requiring mycoplasmas (Vanden Bossche et al., 1993; Marichal et al., 1999). This combined effect completely blocks not only ergosterol synthesis but also that of 4,4-demethylated 14α-methylsterols. This is the first report of inhibition of an NADPH-dependent 3-ketosteroid reductase by a sterol hydrazone derivative.

It is likely that the first metabolic step in the 24-alkyl sterol synthetic pathway of *Sporothrix* sp. involves C24-methylation of lanosterol to yield eburicol, as has been described in studies of the ergosterol pathway of the filamentous ascomycete *Aspergillus fumigatus* (Alcazar-Fuoli et al., 2008) and of the basidiomycete *Cryptococcus neoformans* (Nes et al., 2009; Nes, 2011). The 24-SMTs of *Sporothrix* sp. were significantly more sensitive to the action of H3 than to that of itraconazole. The former is associated with a stronger antiproliferative effect by means of a complete blockage of ergosterol biosynthesis.

SEM and TEM analyses corroborated MIC data showing that *S. schenckii* yeast was more sensitive to the effects of either of the two drugs (H3 and itraconzaole) when compared to *S. brasiliensis* (**Figures 3** and **4**). Both treatments induced different changes depending on the species. In *S. schenckii* 4821 treatment with H3 lead to an increased in cell size, alterations in cell shape, and cytoplasmic electron-density reduction. In contrast, *S. brasiliensis* 4823 only showed alteration in cell wall thickness, with a normal cell size and shape. Alterations in thickness of cell wall components was also observed after treatment of *C. albicans* with other 24-SMT inhibitors (Ishida et al., 2009). Considering itraconazole treatment, both species also react in a different way. *S. schenckii* 4821, treatment induced yeast filamentation, decreased in cell size and disarrangement of cellular compartments (as Golgi complex and vacuoles) and cell wall structure. On the other hand, *S. brasiliensis* 4823 exposure to itraconazole showed an increase in cell size, reduction on cytoplasmic electron-density and alteration in cell wall thickness. Regarding to morphological alterations in *S. schenckii* and *S. brasiliensi*, we can say that membrane plasmatic of these fungi have special requirement for ergosterol, in order to maintain both membrane integrity and fluidity, amounts others possible functions. Therefore, compounds that could affect ergosterol homeostasis like of H3 or itraconazole, may generate (i) loss of these important physical characteristics, (ii) accumulation of endogenous intermediates such as lanosterol or 14α-methy-sterol. Both effects produce different and complex morphologies, which are difficult to correlate at the first moment.

Flow cytometry analyzes showed that H3 induced mitochondrial disturbances in *Sporothrix* sp.; however, while H3-treated *S. schenckii* 4821 cells showed an increased fluorescence intensity when stained with MitoTracker Red CMXRos; *S. brasiliensis* 4823 cells exhibited a decreased fluorescence intensity to the same markers (**Figure 5**). The accumulation of different ergosterol intermediates that could be toxic themselves for the cells could help to explain these differences observed between the two species.

We also investigated whether concurrent treatment with H3 could enhance the antifungal effect of itraconazole, since they inhibit different steps of the ergosterol biosynthesis pathway. A checkerboard assay showed that the two antifungals had a synergistic effect against the *S. brasiliensis* isolates 4823 and Ss37, and that there was a reduction of itraconazole MICs for all other tested isolates despite the lack of statistically significant synergy (**Table 3**), showing that H3 can enhance the anti-*Sporothrix* effectiveness of itraconazole. These results suggest that treatment of sporotrichosis with a combination of these two drugs may be of value. Synergistic effects between H3 and another azole compound (posaconazole) were previously reported in *C. neoformans* (Vivas et al., 2011).

In addition, cytotoxicity assays revealed that H3 had no hemolytic activity toward human red blood cells (at concentrations of up to 100 mg/L) and had low cytotoxicity toward LLCMK-2 cells (at a concentration of 100 mg/L) (**Table 4**). The cytotoxicity results, combined with the high sensitivity of *S. schenckii* and *S. brasiliensis* to H3, mean that H3 has higher selectivity toward *Sporothrix* sp. than does itraconazole.

The results of this preliminary study showing the effects of H3 *in vitro* indicate that this molecule can potentially be used for the development of improved treatments for sporotrichosis. More studies should be conducted to confirm the activity of this drug *in vivo*.

In summary, inhibition of Δ^24^-sterol methyltransferase (24-SMT) by the sterol hydrazone analog H3 was an effective antifungal strategy against *S. schenckii* and *S. brasiliensis*, being more potent than itraconazole in inhibiting ergosterol synthesis. H3 was also more selective toward fungal cells than was itraconazole, and was able to enhance the anti-*Sporothrix* effectiveness of itraconazole when used in conjunction with that drug. Inhibition of the methylation reaction catalyzed by 24-SMT has a strong antiproliferative effect via disruption of ergosterol homeostasis, suggesting that this enzyme is a promising target for novel antifungal therapies against sporotrichosis, either as sole treatments or in combination with itraconazole.

## Author Contributions

GV, KI, WS, and SR designed and coordinated the study. LB-S, GV, and TB carried out experiments. LB-S, GV and SR drafted the manuscript. AR, ZC and LL-B provided *Sporothrix* sp. isolates used in this work and helped to draft the manuscript. All authors read, contributed and approved the final manuscript.

## Conflict of Interest Statement

The authors declare that the research was conducted in the absence of any commercial or financial relationships that could be construed as a potential conflict of interest.
